# Unawareness of breast cancer family history among African women

**DOI:** 10.11604/pamj.2023.45.188.21616

**Published:** 2023-08-30

**Authors:** Babatunde Adedokun, Adeyinka Ademola, Timothy Makumbi, Stella Odedina, Imaria Agwai, Paul Ndom, Antony Gakwaya, Temidayo Ogundiran, Oladosu Ojengbede, Dezheng Huo, Olufunmilayo I. Olopade

**Affiliations:** 1 Center for Clinical Cancer Genetics and Global Health, University of Chicago, Chicago, United States of America,; 2 Department of Surgery, University of Ibadan, Ibadan, Nigeria,; 3 Department of Surgery, Mulago Hospital, Kampala, Uganda,; 4 Center for Population and Reproductive Health, University of Ibadan, Ibadan, Nigeria,; 5 Hôpital Général Yaoundé, Yaoundé, Cameroon,; 6 School of Medicine, St. Augustine International University, Kampala, Uganda

**Keywords:** Family history, familial cancer risk, breast cancer, sub-Saharan Africa

## Abstract

**Introduction:**

comprehensive cancer risk assessment services are lacking in most sub-Saharan African countries and the use of accurate family history (FH) information could serve as a cheap strategy for risk evaluation. The aim of this study is to determine the proportion of women unaware of family history of cancer among female relatives and associated socio-demographic characteristics.

**Methods:**

using case-control data on breast cancer among 4294 women in Nigeria, Uganda and Cameroon, we investigated the proportion of women unaware of family history of cancer among their female relatives. The association between participants' response to their awareness of female relatives' cancer history and socio-demographic characteristics was analysed according to case-control status, family side and distance of relation. **Results:** the proportion of women unaware if any relative had cancer was 33%, and was significantly higher among controls (43.2%) compared to 23.9% among cases (p<0.001) (Adjusted Odds Ratio (OR) = 2.51, 95% CI = 2.14 - 2.95). Age, education and marital status remained significantly associated with being unaware of FH among controls on multiple regression.

**Conclusion:**

about a third of women interviewed did not know about cancer history in at least one of their female relatives. Efforts aimed at improving cancer awareness in sub-Saharan Africa (SSA) are needed. Our findings could be useful for future studies of cancer risk assessment in SSA.

## Introduction

Family history (FH) is an important tool in clinical practice with utility in the diagnosis and management of single-gene disorders and chronic diseases. It also plays an important role in public health as a tool for risk assessment, decision-making about tailored interventions, and as a motivation for behaviour change [1]. In the context of cancer prevention and treatment, family history is an invaluable tool in the management of hereditary cancers including the assessment of risk and decision-making about surveillance or preventive treatment. For example, the US Preventive Services Task Force recommends primary care providers routinely collect and update family medical history and screen women with a family history of breast, ovarian, tubal, or peritoneal cancer to determine if there is a need for in-depth genetic counseling to consider BRCA testing [2]. Moreover, family history is included as a main component of risk predictor models for the evaluation of breast cancer risk or the risk of carrying deleterious mutations in high susceptibility genes such as BRCA [3].

The accuracy of family history reports has been the focus of several investigations with varying reports about accuracy [4-7]. Additionally, There has been a focus on the ease of use of FH tools [8], clinicians' knowledge [9] and ability to elicit and document complete FH information [10]. The role of families in providing accurate FH information cannot be overemphasised. Some studies have shown aspects of family health history communication to be more challenging among African Americans and immigrant populations. Other studies reveal different patterns of FH communication and family tensions [11] while challenges could exist in communicating breast cancer FH across generations such as from mothers to daughters [12] with knowledge about cancer influencing communication with daughters in others [13].

Although a small proportion of breast cancer is hereditary (about 5-10%), life-saving interventions now exist for individuals with hereditary breast cancer and their relatives. The risk of breast cancer is about 2 times higher for women with one affected first-degree female relative and 3-4 times higher for women with more than one first-degree relative compared to women without a family history [14]. The most significant genes implicated in hereditary breast cancer are the BRCA1 and BRCA2 genes, and pathogenic mutations in these genes together accounting for about 30% of high-risk breast cancer families and explain about 15% of the breast cancer familial relative risk [15,16]. Multi-gene panel testing is now available for breast cancer susceptibility genes, enabling the implementation of preventive interventions and personalized medicine [17].

While the management of familial breast cancer is well established in the developed world, with the availability of cancer risk clinics for the evaluation of breast cancer patients and their families, the situation is different in developing countries where these services are not available in most health institutions. Moreover, in the SSA region, breast cancer is disproportionately of the aggressive triple-negative type with late presentation and poor prognosis. Additionally, we have recently reported a high prevalence of deleterious BRCA mutations among Nigerian women [18,19], supporting the argument for the provision of services for the management of hereditary breast cancer in low resource settings such as SSA. In most SSA countries where comprehensive cancer risk assessment services are lacking and genetic testing is not available, use of accurate FH information could serve as a cheap strategy for the evaluation and decision-making for women with breast cancer family history [20]. Such evaluations will require accurate and complete FH information, however, data on the quality of FH reports, best modes of obtaining FH data, health worker capacity, and knowledge concerning history taking/interviews about FH in SSA are lacking. Using data from the African Breast Cancer Study (ABCS), a case-control study of breast cancer among sub-Saharan African women in Nigeria, Uganda, and Cameroon, we determined the proportion of women unaware of family history of cancer among female relatives and socio-demographic characteristics that could influence FH reports.

## Methods

**Study design:** data for this study was obtained from the African Breast Cancer Study (ABCS), a case-control study of the risk factors for breast cancer in Nigeria, Cameroon, and Uganda. The study protocol was reviewed by the institutional review boards of the three sites and the University of Chicago Biological Sciences Division Institutional Review Board (13304B and 10-023-B), University of Ibadan/University College Hospital Ethics Committee (UI/IRC/02/0003), Cameroon National Ethics Committee (N141/CNE/SE/2010), and Makerere University College of Health Sciences Ethics Committee (2011-023). Details of the study methods are provided in previous publications [21].

**Participants:** briefly, women aged 18 years and above with breast cancer and age-matched controls without breast cancer in Nigeria (since 1999) and in Uganda and Cameroon (since 2005) were recruited from tertiary hospitals in the three countries. Cases were defined as females who were 18 years or older, black of African descent, capable of providing informed consent, and had a histologic or clinical diagnosis of invasive breast cancer while controls were those of the same age criteria, without breast cancer, and able to give informed consent.

**Data sources:** face-to-face interviews were conducted to obtain data on socio-demographic characteristics and lifestyle variables, anthropometric characteristics, previous treatments for breast conditions, and family history of cancer. We investigated the proportion of women unaware of family history of cancer among their female relatives (mother, paternal and maternal grandmother, full and half-sisters, father's full and half-sisters, mother's full and half-sisters, and daughters). Data on the response to the question about a family history of cancer among female relatives was analysed as the dependent variable. The proportion of participants that responded ‘don't know’ to any of the female relatives was determined and analysed according to case-control status, family side (father- or mother-side), and distance of relation.

**Statistical methods:** Generalized Estimating Equations (GEE) with the logistic regression option were used to compare the odds of reporting don't know responses for female relatives, adjusting for socio-demographic characteristics. Multiple logistic regression analysis was used to determine socio-demographic factors associated with ‘don't know’ response for any of the female relatives.

## Results

### Socio-demographic characteristics

Of 4294 women, there were 2271 cases and 2023 controls; 450 were studied in Uganda (231 cases and 219 controls), 570 in Cameroon (297 cases and 273 controls), and 3335 in Nigeria (1743 cases and 1531 controls). The mean age of cases was 48.5 years (SD = 11.9) compared to 44.4 years (SD = 13.0) for controls. Table 1 shows the distribution of socio-demographic characteristics by country and case-control status. The predominant ethnic groups were the Yoruba in Nigeria, the Baganda in Uganda, and the Bantous and Semi-Bantous in Cameroon. Overall, about a quarter of cases and one-fifth of controls had attained tertiary education, lower in Cameroon (about 15%) and Uganda (8%) compared to Nigeria (26% of cases and 44% of controls). Almost three-quarters of participants were currently married, much higher in Nigeria compared to Uganda and Cameroon. Concerning parity, the highest proportion of women had 5 or more children (42%), the highest in Uganda. Knowledge of female relative's cancer history: The proportion of women unaware if any relative had cancer was 33%, and was significantly higher among controls (43.2%) compared to cases (23.9%) (p<0.001) (Table 2). The highest proportion of women unaware of cancer FH in female relatives gave such response concerning their grandparents. The proportion reporting DK was significantly higher among controls except for reports for cancer history in daughters. On the maternal side of the family, 19.9% (14.4% of cases and 26.2% of controls) reported don't know, while on the paternal side, 25.5% was unaware of family history (17% among cases and 35.2% among controls). After adjusting for socio-demographic characteristics and the number of female relatives, cases were more likely than controls to answer ‘don't know’ to questions about cancer in a relative except when that relative was a daughter (Table 2). Overall, controls were about 2.5 times more likely than cases to report they did not know cancer FH in female relative (95% CI = 2.14 - 2.95).

**Table 1:** socio-demographic characteristics of women studied

Variable	Nigeria		Uganda		Cameroon		All	
	**Cases (n=1743)**	**Controls (n=1531)**	**Cases (n=231)**	**Controls (n=219)**	**Cases (n=297)**	**Controls (n=273)**	**Cases (n=2271)**	**Controls (n=2023)**
**Age**								
<30	45(2.6)	220(14.4)	16(6.9)	19(8.7)	11(3.7)	10(3.7)	72(3.2)	249(12.4)
30-39	352(20.4)	421(27.6)	53(22.9)	42(19.3)	71(23.9)	69(25.4)	476(21.1)	532(26.4)
40-49	577(33.4)	377(24.7)	59(25.5)	79(33.5)	88(29.6)	87(32.0)	724(32.1)	537(26.6)
50-59	409(23.7)	282(18.5)	59(25.5)	55(25.2)	85(28.6)	69(25.4)	553(24.5)	406(20.1)
60+	346(20.0)	227(14.9)	44(19.1)	29(13.3)	42(14.1)	37(13.6)	432(19.1)	293(14.5)
**Ethnicity**								
**Nigeria**								
Yoruba	1356(77.9)	1163(76.1)					1404(78.0)	1167(57.1)
Ibo	225(12.9)	82(5.4)					230(12.8)	82(5.3)
Hausa	6(0.3)	171(11.2)					6(0.3)	171(11.1)
Other Nigeria	156(8.9)	112(7.3)					156(8.7)	112(7.3)
**Uganda**								
Baganda			101(44.5)	97(44.9)			113(44.7)	107(45.2)
Other Uganda			126(55.5)	119(55.1)			135(53.4)	127(53.6)
**Cameroon**								
Bantous					150(51.2)	123(45.4)	150(50.3)	123(45.1)
Semi bantous					125(42.7)	142(52.4)	125(42.0)	142(52.0)
Other Cameroon					18(6.1)	6(2.2)	18(6.0)	6(2.2)
**Education**								
None	264(15.2)	217(14.6)	25(11.1)	12(5.7)	8(2.7)	3(1.1)	297(13.2)	232(11.8)
Primary	398(23.0)	467(31.5)	99(43.8)	97(45.8)	87(29.8)	68(25.3)	584(25.9)	632(32.2)
Secondary	453(26.1)	381(25.7)	66(29.2)	52(24.5)	137(46.9)	148(55.0)	656(29.1)	581(29.6)
Vocational/Technical	110(6.3)	78(5.3)	18(8.0)	34(16.0)	15(5.1)	13(4.8)	143(6.4)	125(6.4)
Degree/PG	509(29.4)	340(22.9)	18(8.0)	17(8.0)	45(15.4)	37(13.8)	572(25.4)	394(20.1)
**Religion**								
Christianity	1290(74.5)	853(56.0)	214(93.9)	188(88.3)	274(93.2)	267(98.5)	1778(78.9)	1308(65.2)
Islam	441(25.5)	670(44.0)	14(6.1)	25(11.7)	20(6.8)	4(1.5)	475(21.1)	699(34.8)
**Marital status**								
Married	1278(73.5)	1231(80.6)	117(50.7)	128(58.7)	162(54.6)	133(48.7)	1557(68.7)	1492(73.9)
Widowed	291(16.7)	169(11.1)	38(16.5)	32(14.7)	52(17.5)	38(13.9)	381(16.8)	239(11.8)
Divorced	54(3.1)	10(0.7)	8(3.5)	7(3.2)	8(2.7)	9(3.3)	70(3.1)	26(1.3)
Separated	48(2.8)	25(1.6)	51(22.1)	34(15.6)	8(2.7)	9(3.3)	107(4.9)	68(3.4)
Never married	67(3.9)	92(6.0)	17(7.4)	17(7.8)	67(22.6)	84(30.8)	151(6.7)	193(9.6)
**Number of livebirths**								
<3	364(22.6)	348(24.3)	49(23.7)	50(24.0)	88(32.4)	80(31.6)	501(24.0)	478(25.3)
3-4	583(36.2)	493(34.5)	41(19.8)	56(26.9)	82(30.2)	66(26.1)	706(33.8)	615(32.5)
5+	664(41.2)	590(41.2)	117(56.5)	102(49.0)	102(37.5)	107(42.3)	883(42.3)	799(42.2)

**Table 2:** comparison of 'don't know' responses to cancer history in female relative between cases and controls and by relative type

	Bi-variable analysis										Multivariable analysis**
	**All**			**Cases**			**Controls**				
					
**Relative type**	**% any DK**	**Number of relatives median (min, max)**	**N**	**% any DK**	**Median number of relatives (min, max)**	**N**	**% any DK**	**Median number of relatives (min, max)**	**N**	P value*	**OR (95% CI)**
Mother	4.9	1	4265	4.3	1	2253	5.6	1	2012	**0.041**	**1.41 (1.02 - 1.96)**
Maternal grandmother	16.3	1	3993	12.2	1	1983	20.4	1	2010	**<0.001**	**2.39 (1.96 - 2.91)**
Paternal grandmother	24.0	1	3799	17.2	1	1818	30.3	1	1981	**<0.001**	**2.70 (2.26 - 3.23)**
Sister	7.0	3(1, 32)	3928	5.7	2(1,10)	2030	8.4	3(1,32)	1898	**0.001**	**1.47 (1.10 - 1.97)**
Mother’s sister	12.2	2(1, 18)	3190	8.7	2(1,9)	1581	15.5	2(1,18)	1609	**<0.001**	**2.13 (1.66 - 2.76)**
Father’s sister	14.9	2(1, 15)	2737	10.8	1(1,6)	1282	18.5	2(1,15)	1455	**<0.001**	**2.22 (1.71 - 2.87)**
Daughter	2.8	2 (1, 9)	3369	2.7	2(1,9)	1761	2.9	2(1,9)	1608	0.655	1.02 (0.66 - 1.60)
Any female relative	33.0	10(1, 41)	4294	23.9	9(1,25)	1761	43.2	11(1,41)	1608	**<0.001**	**2.51 (2.14 - 2.95)**

* Based on chi square tests comparing proportions of women reporting any ‘don’t know’ (DK) between cases and controls for each relative type **ORs comparing ‘don’t know’ responses between controls with cases, adjusted for age, education, religion, marital status, parity, ethnicity, and number of relative type (the model for ‘any female relative’ was adjusted for total number of female relatives) Significant results are in bold print

### Knowledge of cancer in female relative and associated factors

Table 3 and Table 4 show the association between awareness of cancer history in female relatives and socio-demographic variables, for cases and controls and by study site. In both cases and controls, Uganda had a significantly higher proportion of women that answered they were unaware of female relatives' FH of cancer, compared to Cameroon and Nigeria. Among cases, these responses were significantly higher among Christians, those not currently married, and women with lower education (p<0.001 for the three associations). There was also a significant association for a number of live births (Table 3). These significant associations among all cases were not significant for the three countries studied except for educational status in Cameroon. Among controls, the proportion of women unaware of cancer FH in female relatives was higher among older women, those not currently married, and Christians; and significantly differed by ethnic group in Nigeria and Cameroon (Table 4). The direction of the associations for education differed between countries; responses of ‘don't know’ were higher among women with higher education in Nigeria, but the highest proportion was among those with no formal education in Uganda. In Uganda, higher proportions unaware of FH were found among women with more children, but this association was not found in Cameroon or Nigeria. Unmarried Cameroonian controls significantly reported they were unaware of female relative cancer FH compared to their married counterparts (Table 4). On multiple logistic regression, Ugandan cases and controls were significantly more likely to report they were unaware of cancer FH compared to women in Nigeria and Cameroon (Table 5, Table 6). Significantly higher odds ratios were found for comparisons of DK responses for all female relatives compared to cancer history in mothers except for sisters. The pattern of observed significant differences was similar for the three countries except for Cameroon. Additionally, the direction of the association was different for sister-mother comparisons with higher odds ratios among Nigerian women but lower for Ugandans. Concerning ethnicity, among controls, women of the Ibo ethnic group were more likely than Yoruba to report DK, while Semi-Bantous in Cameroon were also more likely than the Bantous to be unaware of cancer FH. Age and education remained significantly associated with being unaware of FH among Nigerian women and overall. For educational status, women with higher education in Nigeria remained more likely, while those with secondary or vocational education in Uganda were less likely compared to women with lower education to be unaware of cancer FH. Overall, unmarried women were more likely than those currently married to be unaware of cancer FH in female relatives (Table 6).

**Table 3:** association between 'don't know' responses and socio-demographic characteristics among cases and by country

Variable	Nigeria		Uganda		Cameroon		All	
	**%**	**N**	**%**	**N**	**%**	**N**	**%**	**N**
**Age**								
<30	13.3	45	***	16	***	11	31.9	72
30-39	16.5	352	58.5	53	49.3	71	26.1	476
40-49	14.6	577	64.4	59	36.4	88	21.3	724
50-59	14.7	409	62.7	59	45.9	85	24.6	553
60+	17.1	346	65.9	44	33.3	42	23.6	432
**P value**		0.798		0.955		0.163		0.153
**Ethnicity**								
**Nigeria**								
Yoruba	14.5	1356						
Ibo	18.7	225						
Hausa	***	6						
Other Nigeria	18.8	154						
**P value**		0.141						
**Uganda**			67.3	101				
Baganda			59.5	126				
Other Uganda				0.226				
**P value**								
**Cameroon**					40.0	150		
Bantous					47.2	125		
Semi bantous					27.8	18		
Other Cameroon						0.211		
**P value**								
**Education**								
None/Primary	16.8	662	62.1	124	35.8	95	25.2	881
Secondary/Vocational	16.0	563	63.1	84	51.3	152	27.7	799
Degree/PG	13.2	509	72.2	18	31.1	45	16.4	572
**P value**		0.219		0.706		**0.012**		**<0.001**
**Religion**								
Christianity	15.0	1290	63.6	214	43.8	274	24.0	1833
Islam	16.3	441	***	14	25.0	20	15.9	496
**P value**		0.517		0.310		0.101		**<0.001**
**Currently married**								
Yes	14.9	1278	59.0	117	41.4	162	20.9	1557
No	17.2	460	66.7	114	44.4	135	30.3	709
**P value**		0.241		0.227		0.592		**<0.001**
**Number of livebirths**								
<3	18.4	364	59.2	49	36.4	88	25.6	501
3-4	13.4	583	58.5	41	47.6	82	20.0	706
5+	16.9	664	64.1	117	44.1	102	26.3	883
**P value**		**0.085**		0.745		0.313		**0.009**
**Site**								
Nigeria							15.5	1743
Uganda							62.8	231
Cameroon							42.8	297
**P value**								**<0.001**

*** Suppressed due to small sample size (<20)

**Table 4:** association between 'don't know' responses and socio-demographic characteristics among controls and by country

Variable	Nigeria		Uganda		Cameroon		All	
	**%**	**N**	**%**	**N**	**%**	**N**	**%**	**N**
**Age**								
<30	30.9	220	***	19	***	10	32.5	249
30-39	37.3	421	69.1	42	42.0	69	40.4	532
40-49	45.1	377	67.1	73	35.6	87	46.6	537
50-59	44.3	282	74.6	55	47.8	69	49.0	406
60+	40.1	227	79.3	29	35.1	37	43.3	293
P value		**0.004**		**0.023**		0.354		**0.001**
**Ethnicity**								
**Nigeria**								
Yoruba	38.1	1163						
Ibo	61.0	82						
Hausa	30.4	171						
Other Nigeria	58.9	112						
**P value**		**<0.001**						
**Uganda**								
Baganda			71.1	97				
Other Uganda			65.6	119				
**P value**				0.381				
**Cameroon**								
Bantous					32.5	123		
Semi bantous					47.2	142		
P value						**0.015**		
**Education**								
None/Primary	36.1	684	78.9	109	47.9	71	42.5	864
Secondary/Vocational	44.7	459	53.5	86	38.5	161	44.3	706
Degree/PG	46.8	340	70.6	17	40.5	37	47.2	394
P value		**0.001**		**0.001**		0.407		0.289
**Religion**								
Christianity	43.7	853	67.0	188	41.6	267	45.6	1324
Islam	35.2	670	76.0	25	***	4	35.7	705
P value		**0.001**		0.366		0.504		**<0.001**
**Currently married**								
Yes	39.9	1231	68.8	128	34.6	133	41.9	1492
No	40.5	296	67.8	90	47.1	140	47.0	526
P value		0.837		0.879		**0.035**		**0.044**
**Number of live births**								
<3	38.8	348	58.0	50	41.3	80	41.2	478
3-4	41.8	493	62.5	56	34.9	66	42.9	615
5+	40.7	590	78.4	102	44.9	107	46.1	799
P value		0.684		**0.017**		0.429		0.208
**Site**								
Nigeria							40.0	1531
Uganda							68.0	219
Cameroon							41.0	273
P value								**<0.001**

*** Suppressed due to small sample size (<20)

**Table 5:** multiple logistic regression of factors associated with 'don't know' responses among cases

Variable	Nigeria		Uganda		Cameroon		All		
	**0R**	**95% CI**	**0R**	**95% CI**	**0R**	**95% CI**	**0R**	**95% CI**	
**Age**									
<30	1		1		1		1		
30 - 49	0.94	0.26 - 3.39	0.58	0.17 - 2.03	0.36	0.08 - 1.62	0.72	0.34 - 1.55	
50+	1.18	0.32 - 4.37	0.99	0.27 - 3.57	0.76	0.17 - 3.48	1.09	0.49 - 2.40	
**Ethnicity**									
**Nigeria**									
Yoruba	1						1		
Ibo	1.49	0.97 - 2.27					1.52	0.99 - 2.36	
Hausa	1.36	0.22 - 8.47					2.40	0.35 - 16.19	
Other Nigeria	1.71	1.02 - 2.85					1.61	0.96 - 2.71	
**Uganda**									
Baganda			1				0.72	0.96 - 1.88	
Other Uganda			0.68	0.39 - 1.19					
**Cameroon**									
Bantous					1		0.91	0.56 - 1.45	
Semi bantous					1.16	0.69 - 1.96	***	***	
**Education**									
None/Primary	1		1		1		1		
Secondary/Vocational	0.91	0.63 - 1.30	0.78	0.43 - 1.42	1.79	0.95 - 3.38	1.05	0.81 - 1.37	
Degree/PG	0.69	0.45 - 1.05	0.70	0.21 - 2.36	0.54	0.18 - 1.61	0.64	0.45 - 0.92	
**Religion**									
Christianity	1		1		1		1		
Islam	1.36	0.94 - 1.95	0.11	0.001 - 17.49	0.48	0.08 - 3.13	1.06	0.75 - 1.50	
**Currently married**									
Yes	1		1		1		1		
No	1.12	0.79 - 1.59	0.58	0.32 - 1.03	0.67	0.38 - 1.18	0.87	0.67 - 1.14	
**Number of live births**									
<3	1		1		1		1		
3-4	0.73	0.49 - 1.09	1.10	0.41 - 2.94	1.43	0.73 - 2.83	0.85	0.62 - 1.17	
5+	0.76	0.50 - 1.16	1.35	0.61 - 2.96	1.01	0.49 - 2.10	0.89	0.65 - 1.22	
**Relative type**									
Mother	1		1		1		1		
Motherï¿½s mum	**3.96**	**2.68 - 5.87**	**2.41**	**1.64 - 3.53**	**3.82**	**2.35 - 6.19**	**3.19**	**2.55 - 4.00**	
Fatherï¿½s mum	**5.87**	**4.00 - 8.63**	**3.46**	**2.30 - 5.19**	**6.23**	**3.85 - 10.08**	**4.78**	**3.82 - 5.99**	
Sister	1.31	0.84 - 2.04	**0.59**	**0.36 - 0.97**	0.70	0.42 - 1.16	0.85	0.65 - 1.12	
Motherï¿½s sister	**2.79**	**1.77 - 4.39**	1.09	0.70 - 1.71	1.36	0.82 - 2.25	**1.65**	**1.27 - 2.15**	
Fatherï¿½s sister	**3.00**	**1.88 - 4.81**	**1.92**	**1.21 - 3.07**	**1.87**	**1.13 - 3.09**	**2.30**	**1.76 - 3.01**	
Daughter	**0.41**	**0.22 - 0.78**	**0.43**	**0.23 - 0.79**	0.57	0.30 - 1.08	**0.48**	**0.33 - 0.68**	
**Site**									
Uganda							1		
Nigeria							**0.50**	**0.31 - 0.80**	
Cameroon							**0.12**	**0.08 - 0.18**	

*** Omitted from the model due to multicollinearity

**Table 6:** multiple logistic regression of factors associated with 'don't know' responses among controls

Variable	Nigeria		Uganda		Cameroon		All	
	**0R**	**95% CI**	**0R**	**95% CI**	**0R**	**95% CI**	**0R**	**95% CI**
**Age**								
<30	1		1		1		1	
30 - 49	1.44	0.95 - 2.20	3.24	0.86 - 12.23	0.69	0.11 - 4.12	**1.54**	**1.02 - 2.32**
50+	**1.91**	**1.17 - 3.15**	**5.22**	**1.33 - 20.42**	0.50	0.08 - 3.20	**1.91**	**1.19 - 3.05**
**Ethnicity**								
**Nigeria**								
Yoruba	1						1	
Ibo	**2.86**	**1.84 - 4.43**					**2.83**	**1.82 - 4.41**
Hausa	1.21	0.72 - 2.04					1.01	0.62 - 1.66
Other Nigeria	**2.24**	**1.58 - 3.16**					**2.21**	**1.55 - 3.14**
**Uganda**								
Baganda			1				1.05	0.62 - 1.79
Other Uganda			0.81	0.46 - 1.41				
**Cameroon**								
Bantous					1		0.36	0.13 - 0.99
Semi bantous					**2.69**	**1.42 - 5.09**	***	***
Other Cameroon							***	***
**Education**								
None/Primary	1		1		1		1	
Secondary/Vocational	**1.41**	**1.04 - 1.91**	**0.53**	**0.28 - 0.99**	0.72	0.34 - 1.56	1.15	0.87 - 1.50
Degree/PG	**1.58**	**1.13 - 2.19**	0.80	0.27 - 2.40	0.92	0.30 - 2.75	**1.91**	**1.19 - 3.05**
**Religion**								
Christianity	1		1		1		1	
Islam	0.82	0.60 - 1.11	1.14	0.46 - 2.82	0.64	0.60 - 6.83	0.82	0.63 - 1.07
**Currently married**								
Yes	1		1		1		1	
No	1.53	1.10 - 2.16	1.33	0.77 - 2.31	2.56	1.28 - 5.13	**1.60**	**1.21 - 2.11**
**Number of livebirths**								
<3	1		1		1		1	
3-4	1.11	0.80 - 1.53	0.74	0.32 - 1.69	1.37	0.58 - 3.24	1.09	0.82 - 1.45
5+	0.97	0.68 - 1.38	0.73	0.35 - 1.52	2.98	1.22 - 7.25	1.07	0.79 - 1.46
**Relative type**								
Mother	1		1		1		1	
Mother’s mum	**7.26**	**5.34 - 9.86**	**4.36**	**2.91 - 6.52**	**2.35**	**1.60 - 3.44**	**5.02**	**4.10 - 6.14**
Father’s mum	**13.40**	**9.77 - 18.40**	**6.36**	**4.16 - 9.73**	**4.79**	**3.16 - 7.25**	**9.07**	**7.36 - 11.19**
Sister	**1.54**	**1.06 - 2.23**	**0.53**	**0.32 - 0.88**	1.28	0.87 - 1.87	1.14	0.90 - 1.46
Mother’s sister	**3.64**	**2.56 - 5.18**	**2.35**	**1.54 - 3.57**	**1.47**	**1.05 - 2.06**	**2.68**	**2.14 - 3.36**
Father’s sister	**5.47**	**3.92 - 7.63**	**2.03**	**1.29 - 3.16**	1.42	0.93 - 2.16	**3.49**	**2.79 - 4.37**
Daughter	**0.19**	**0.08 - 0.44**	**0.16**	**0.07 - 0.38**	**0.54**	**0.31 - 0.94**	**0.27**	**0.18 - 0.42**
**Site**								
Uganda							1	
Nigeria							**0.21**	**0.11 - 0.41**
Cameroon							**0.26**	**0.17 - 0.40**

* For ethnicity, ‘Other Cameroon’ and ‘Other Uganda’ were excluded from model for overall due to multicollinearity

## Discussion

About a third of women interviewed in a breast cancer case-control study setting in three African countries did not know about cancer history in at least one of their female relatives. The focus of this study is different from many others that examined the accuracy of family history reports by comparing them with gold standards such as disease registers [4-7]. Rather, we investigated a measure of the level of awareness of women about cancer among their relatives. A high level of awareness of family cancer history and accurate reporting will allow more accurate risk assessments, appropriate breast cancer preventive interventions (such as prophylactic mastectomy and salpingo-oophorectomy and chemotherapy); and motivation for the adoption of cancer risk-reducing behaviour [1]. The proportion of women unaware of cancer history in female relatives' in this study suggests that women in SSA do not pay much attention to the cause of death or diseases affecting their relatives. One implication of this finding is that the estimated family history of breast cancer in the SSA setting could be underestimated, given that some of the ‘don't know’ responses could potentially be 'Yes'. The relatively lower proportions unaware among first-degree relatives such as mothers, sisters and daughters (less than 10%) is expected given that first-degree relatives are more likely to live together and be familiar with one another's illnesses. The proportions unaware of FH were higher for cancer history in grandparents, likely due to the lower level of interaction with older generations because higher generation relatives could have died at relatively younger ages when the women studied were too young to be aware of the cause of death, or not even born and hence never knew about these grandparents. Additionally, awareness about cancer and other causes of death could have improved over the years, being worse in those years when the grandparents lived. Previous studies have shown lower accuracy for family history reports about distant relatives [22,23]. It is crucial that detailed family history for all relatives be obtained especially as important patterns in the family pedigree could guide in more accurate diagnosis, and the importance of complete family history has been emphasised in some studies [24]. Moreover, some risk prediction models for hereditary breast cancer or the risk of carrying a deleterious mutation in BRCA genes require FH of cancer in higher-degree relatives. In addition to the higher rate of DK responses in older relatives, women also reported they were unaware of cancer FH more on the father's side of the family and this finding is supported by studies showed greater bias in cancer family history reports on the father side [22,25].

Significant differences were found between cases and controls in the rates of DK responses with an odds ratio over twice as high among controls compared to cases. The finding could be due to women with breast cancer more willing to answer questions asked in the hospital setting, as they could perceive that providing such information could influence their care. Family history taken during the evaluation of relatives of cancer patients in a cancer-risk clinic setting may be quite different from that in a case-control study, our study setting. The higher DK proportions among controls suggests that cancer-free individuals, including relatives of cancer patients, may need a more vigorous approach in obtaining FH, and interventional studies are required to investigate the efficacy of multiple approaches to obtaining FH. Studies are also needed among relatives of cancer patients that will investigate the rate and accuracy of reporting of cancer FH. Previous studies have shown that educational and motivational messages [26] or the use of mobile technology have significantly improved the quality of cancer FH reports. It is noteworthy that existing FH tools could be challenging for applications in low resource settings such as SSA [8] and innovative, culturally appropriate interventions are required to improve awareness of cancer FH, ability to construct family pedigrees and provide more complete cancer FH information in the SSA setting.

Another explanation for the difference in DK responses between cases and controls is the fact that some of the interviews were conducted at home while others were in the hospital, and the possibility of obtaining information from available family members in a more relaxed atmosphere could improve the quality of the report. On the other hand, more accurate recollection could be possible in the hospital setting as discussed above. It is worthy of note that the lower DK rates among cases do not necessarily imply correct responses for ‘Yes’ and ‘No’ but at least suggest that they make the effort to provide more information. In this study, the DK responses were more likely in Uganda compared to Cameroon and Nigeria, among both cases and controls. We also found differences in DK responses about cancer family history by ethnicity. In Nigeria, Ibos and other ethnic groups compared to Yoruba, and Cameroonian Semi-Bantous compared to Bantous were less aware of cancer family history. The differences found by ethnicity suggest there are possible cultural influences in the level of attention paid to family cancer history in these populations. For example, the Ibos in Nigeria are a more mobile group and could have lower family connectedness, and that could explain their higher DK rates. Future studies using qualitative designs would provide greater insights into the level of awareness of family history of cancer or other common diseases. Previous studies have alluded to the strong cultural influences on responses about FH such as cancer stigmatization or desire to protect relatives [12].

The finding that controls in the oldest age group were more likely to report DK is surprising given that older people should be more familiar with relatives' histories, having been around for a longer period. A similar study about DK responses to a FH of diabetes and heart disease conducted among an underserved minority Mexican population in the United States also found lower, though non-significant levels of unawareness of FH of these diseases among younger people [27]. The higher rates of response to questions about cancer family history among young women in this study could indicate greater curiosity among younger persons or greater cooperation with the interviewer.

Among controls in Nigeria and overall, educated women more commonly gave 'don't know' responses to questions about cancer family history compared to women with primary or no formal education. Among cases, however, the reverse was the case, though only significant in the total sample. The reason for the difference in association between cases and controls is unclear and deserves further study. The limitations of this study include the fact that DK responses may not directly translate to inaccurate responses, and studies of cancer FH accuracy would require study designs that verify FH reports using cancer registry data. However, the findings from this study give some insight into the degree of awareness or interest in cancer FH among native SSA women. Secondly, the controls in this study may not be representative of relatives of cancer patients that constitute the population from which most information about cancer family history would be required for cancer prevention efforts, for example in the cancer risk clinic setting. Additionally, as alluded to earlier, it is also possible that responses from participants with a family history of cancer may be systematically different from those without such history, and studies focusing on relatives of those with breast cancer are advocated. Another limitation is the absence of detailed data on responses to cancer history in male relatives, hence we could not study the rates of awareness of cancer history and compare with our findings for responses about female relatives. In spite of these limitations, we have reported on responses related to family history from a large multi-country SSA sample, and our findings could be useful for future studies of cancer risk assessment in SSA.

**Funding:** This project was supported by Susan G. Komen for the Cure (SAC110026 to O.I. Olopade), NIH Commons Credits Pilot Award (CCREQ-00079 to O.I. Olopade), Breast Cancer Research Foundation (D. Huo, O.I. Olopade), and (R01 CA228198 to D. Huo; U01 CA161032 to O.I. Olopade, D. Huo).

## Conclusion

About a third of women interviewed in three SSA countries did not know about cancer history in at least one of their female relatives. Future studies need to investigate the accuracy of reports of cancer FH, though this could be difficult in the SSA setting where cancer records are incomplete or could be totally absent. The incorporation of precision medicine in patient care in low-resource settings such as SSA may take some more years due to prohibitive costs and a lack of trained personnel. However, a cheap and readily available temporary alternative could be the use of family history data to plan preventive interventions. These findings underscore the need for studies about obtaining family history information in hospital and population settings in sub-Saharan Africa. Research should also investigate health workers as studies have shown only modest knowledge of FH information among medical personnel [9]. In addition to patient perspectives about family history taking, studies are also needed that would examine current family health history-taking practices by physicians and nurses about cancer, documentation, and level of detail of such history. Moreover, data is needed about the best approaches to obtaining family health history information which is particularly important in the cultural or religions settings of SSA countries.

### What is known about this topic

- *The management of familial breast cancer is well established in the developed world unlike in most sub-Saharan African countries;*
- *The use of accurate family history information could serve as a cheap strategy for comprehensive cancer risk assessment in sub-Saharan Africa*.

### What this study adds

- *A third of women did not know about cancer history in their female relatives;*
- *Being unaware of cancer family history was more common among breast cancer cases compared to controls;*
- *Age and education were associated with being unaware of cancer family history*.
